# Supplementary material for: Topological control of chirality and spin with structured light
Source: Light Sci Appl. 2026 Apr 24;15:214. doi: 10.1038/s41377-026-02278-6 (PMC13109355; doi:10.1038/s41377-026-02278-6)
Supplement: Supplementary file 1 — Supplementary Information [file 41377_2026_2278_MOESM1_ESM.pdf]

Topological Control of Chirality and Spin with Structured Light: Supplementary Information

Light Mkhumbuza,[1] Pedro Ornelas,[1] Angela Dudley,[1] Isaac Nape,[1,*] and Kayn A. Forbes[2,†]

[1]*School of Physics, University of the Witwatersrand, Private Bag 3, Wits 2050, South Africa*
[2]*School of Chemistry, University of East Anglia, Norwich Research Park, Norwich NR4 7TJ, United Kingdom*

Evolution of LG modes modulated with azimuthal phases

In the article, we transmit an LG beam initially with the OAM topological charge ℓ_p, $\mathrm{LG}_{\ell_p} = f_{|\ell_p|}(r)\exp(i\ell_p\phi)$ through a q-plate so that each component (A and B) has an OAM charge transfer of $\pm\Delta\ell$ depending on the polarisation component. The output mode then has the field profile, $\mathrm{LG}_{\ell_p}\exp(i\Delta\ell\phi) = f_{|\ell_p|}(r)\exp\big(i\ell_{A(B)}\phi\big)$ where $\ell_{A(B)} = \ell_p \pm \Delta\ell$. It has been found that this beam evolves into an elegant Laguerre Gaussian mode (eLG), having the closed form expression [1],

$$\mathrm{eLG}_p^{\ell_{A(B)}}(\mathbf{r}) = \mathcal{N}\exp\Big[i\big(kz - \tfrac{\pi}{2}(\ell_{A(B)}+1)\big)\Big] \left(\frac{q}{w(z)^2}\right)^{1+p+\frac{|\ell_{A(B)}|}{2}} \exp\left[i\,\frac{k\,r^2}{2\,R(z)}\right] \exp\big[i\,\ell_{A(B)}\,\phi\big]\left(\frac{\sqrt{q}\,r}{w(z)}\right)^{|\ell_{A(B)}|} \exp\left[-\frac{r^2}{w(z)^2}\right] L_p^{|\ell_{A(B)}|}\left[\frac{q\,r^2}{w(z)^2}\right], \quad (1)$$

where

- $w(z) = w_0\sqrt{1+\left(\frac{z}{z_R}\right)^2}$ is the beam waist at distance z.
- $q = 1 + i\,\frac{z_R}{z}$ is the complex beam parameter.
- $R(z) = \frac{z^2+z_R^2}{z}$ is the radius of curvature of the wavefront.
- $z_R = \frac{k\,w_0^2}{2}$ is the Rayleigh range of the embedded Gaussian from the initial $\mathrm{LG}_{\ell_{\mathrm{in}}}$ beam.
- $L_p^{|\ell_{A(B)}|}[\cdot]$ again denotes the generalized Laguerre polynomial.

In Fig. 1 (a) the intensity distribution and 1D cross-section of a pair of eLGs with $\ell_A = 0$ and $\ell_B = 2$ are shown for the plane $\zeta = 0$. Eq. (1), the factor $\big(q/w(z)^2\big)^{1+p+|\ell_{A(B)}|/2}$ and the phase $\exp\big[i\,k\,r^2/(2\,R(z))\big]$ capture the standard Gaussian-beam evolution (spot size, curvature, and Gouy phase).

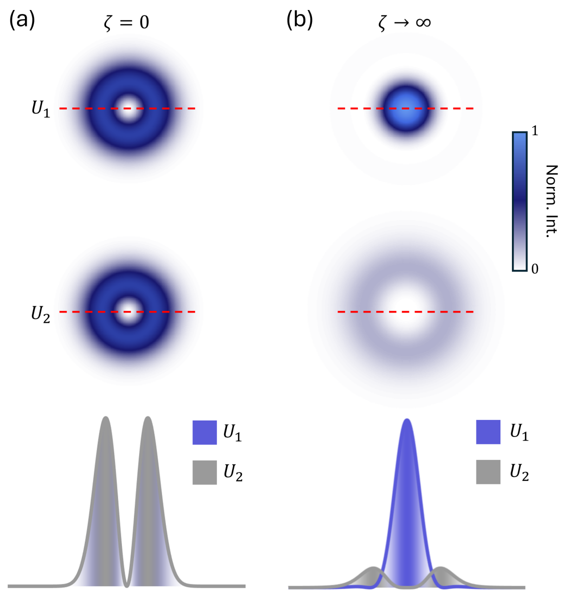

FIG. 1. **Intensity evolution of elegant Laguerre Gaussian modes in propagation to far-field.** Intensity plots for $\mathrm{eLG}_{p=0}^{\ell_A}$ with $\ell_A = 0$ (top panel) and $\ell_B = 2$ (middle panel) along with cross sections of their intensities shown in the bottom panel. Their intensities are show at the (a) $\zeta = 0$ plane and (b) far away from the generation plane $\zeta \to \infty$.

In the far field ($z \gg z_R$), the modified Laguerre–Gaussian field $\mathrm{LG}\,e^{\Delta\ell}$ admits the closed-form expression

$$\Psi^{\mathrm{Far}}_{\ell_{A(B)},p}(\mathbf{k},z) = \frac{\mathcal{N}}{\lambda\,z}\exp\Big[i\big(kz - \tfrac{\pi}{2}(\ell_{A(B)}+1)\big)\Big] \exp\left[i\,\frac{z}{2k}\,k_r^2\right]\left(\frac{k_r\,w}{2}\right)^{|\ell_{A(B)}|} \exp\left[-\left(\frac{k_r\,w}{2}\right)^2\right] L_p^{|\ell_{A(B)}|}\left[\left(\frac{k_r\,w}{2}\right)^2\right] \quad (2)$$

$$\exp\big[i\,\ell_{A(B)}\,\phi\big], \quad (3)$$

where

- $k_r = \frac{k}{z}\,r$ maps the transverse coordinate r to the radial wavenumber.
- The factor $\exp\big[i\,\frac{z}{2k}\,k_r^2\big]$ represents the quadratic (curvature) phase in the far field.
- The term $\exp\big[i\big(kz - \frac{\pi}{2}(\ell_{A(B)}+1)\big)\big]$ combines the longitudinal propagation phase e^{ikz} with the Gouy phase shift $i^{-(\ell_{A(B)}+1)}$.

* isaac.nape@wits.ac.za
† K.Forbes@uea.ac.uk

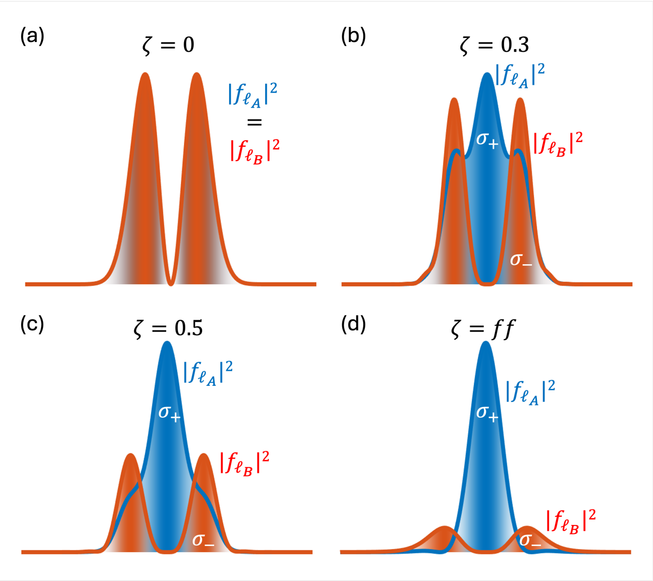

FIG. 2. **Amplitude separation on propagation.** Illustration of the field intensities for the right and left CP components at various longitudinal distances, $\zeta = z/z_R$.

- $L_p^{|\ell_{A(B)}|}[\cdot]$ denotes the generalized Laguerre polynomial of order $(p, |\ell_{A(B)}|)$.

In Fig. 1 (b) the intensity distribution and 1D cross-section of a pair of eLGs with $\ell_A = 0$ and $\ell_B = 2$ are shown for $\zeta \to \infty$. This result is consistent with the asymptotic form of the far-field mode function derived in Saghafi *et al.* [2].

I. SPIN SEPARATION AND VECTOR BEAM SIMULATIONS

In this work, one of the primary mechanisms for the separation of the spin components of the field is the separation of the amplitudes of the field on propagation as shown in Fig. 2. At the plane $\zeta = 0$ where the q-plate phase $e^{i\Delta\ell\phi}$ is applied we have that the amplitudes of the two spin components are identical thus yielding no local separation of the spin components, as shown in Fig. 2 (a). However, immediately after propagation, $\zeta > 0$, the amplitudes start to separate out in space thus yielding a local separation of the spin components as shown in Fig. 2 (b) and (c), achieving a maximal separation as the fields are allowed to propagate very far away from the initial plane, i.e., (d) $\zeta = ff$. In the far-field it is clear that each spin component dominates at different local regions thus yielding a local separation of the spin. This mechanism is further emphasized in Fig. 3 (a) and (b). Here, it is clear that orthogonal spin components emerge immediately after propagation, $\zeta > 0$, shown with the presence of "blue vectors pointing up" and"red vectors pointing down" in Fig. 3 (a) and further all possible polarization states (spin states and every weighted superposition of them) are present within the field for $\zeta > 0$, shown as full coverage over the Poincaré sphere in Fig. 3 (b). A comprehensive set of simulations showing the propagation-evolving Poincaré sphere coverage and spatially-dependent polarization ellipse plots corresponding to the data presented in the main text is shown in Fig. 4 for $\ell_p = -1, 2, -2$. However, in practice it is unlikely that all these polarization states are detectable as some will be found in regions where the intensity is close to the background intensity. The majority of the background noise is isotropic thus after normalization this could lead to erroneous errors in polarization identification in areas of low signal-to-noise ratio. Therefore, in the main text, the simulations are thresholded to match experimental conditions where background noise necessitates noise subtraction, thus appearing as if the orthogonal spin components emerge more gradually.

II. PANCHARATNAM PHASE AND TOPOLOGICAL CHARGE

The topological charge ℓ and azimuthal phase of scalar vortices is well-appreciated. The topological charge describes OAM associated with the scalar vortex beam. The analogous properties of VVBs are the Pancharatnam topological (PT) charge and phase. The Pancharatnam phase of a VVB is given by [3]

$$\phi_p = \arg(\langle \mathbf{E}(\phi = 0)|\mathbf{E}(\phi)\rangle) = \frac{\ell_\mathrm{A} + \ell_\mathrm{B}}{2}\phi, \tag{4}$$

where $|\mathbf{E}(\phi = 0)\rangle$ is taken as a reference. The above phase describes the PT charge of a general VVB given by

$$\mathbf{E} = f_{\ell_\mathrm{A}}(\mathbf{r})\mathrm{e}^{i\ell_\mathrm{A}\phi}\hat{\sigma}_+ + f_{\ell_\mathrm{B}}(\mathbf{r})\mathrm{e}^{i\ell_\mathrm{B}\phi}\hat{\sigma}_-.s \tag{5}$$

Accordingly, the Pancharatnam topological charge is defined as

$$\ell_p = \frac{1}{2\pi}\oint_C \mathrm{d}\phi_p, \tag{6}$$

where the integration path, C, encircles the phase singularity. It is trivial to insert Eq. (4) into Eq. (6) and get the result $\ell_p = (\ell_\mathrm{A} + \ell_\mathrm{B})/2$. In analogous fashion to scalar vortices, the OAM of VVBs is quantified through the Pancharatnam topological charge ℓ_p.

A. Optical chirality and spin angular momentum density of Poincarè beams

The electric field for our vector beam is given as:

$$\mathbf{E} = f_A(\mathbf{r})\mathrm{e}^{i\ell_\mathrm{A}\phi}\hat{\sigma}_+ + f_\mathrm{B}(\mathbf{r})\mathrm{e}^{i\ell_B\phi}\hat{\sigma}_-, \tag{7}$$

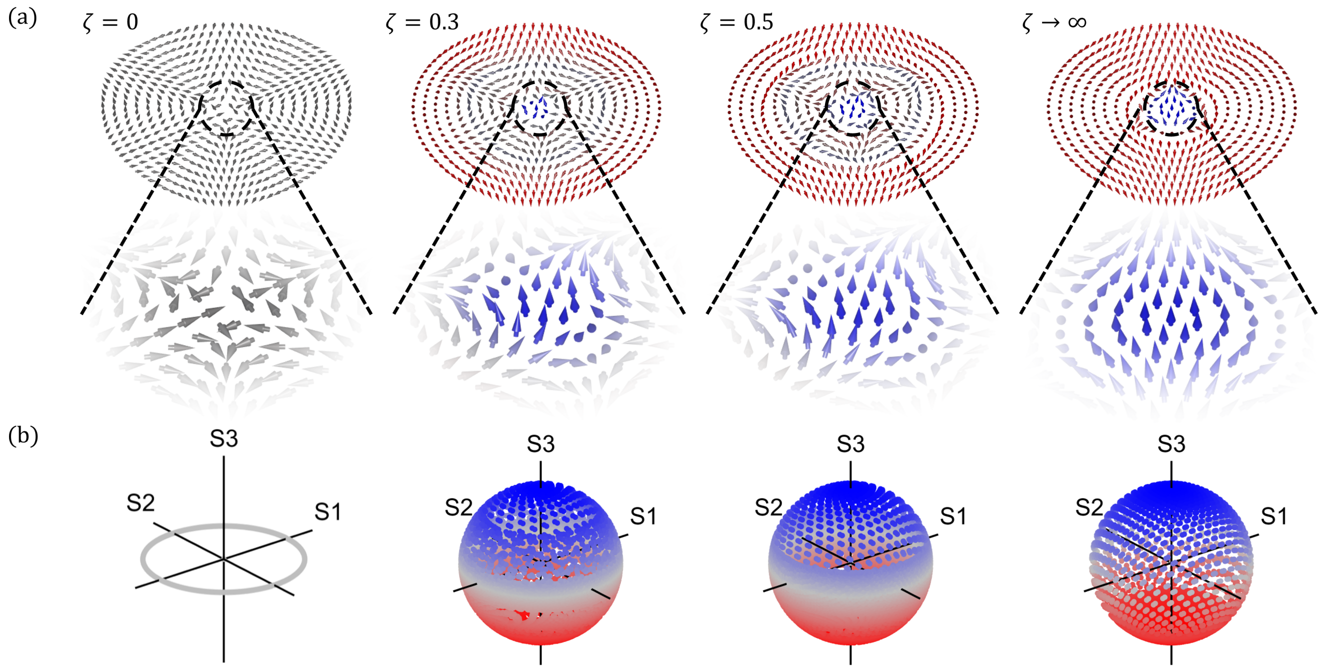

FIG. 3. Extension of Fig. 2 (b) and (c) considering conditions where the entire field intensity can be resolved, demonstrating full coverage over all possible spin states immediately upon propagation. (a) Spin textured fields for the various corresponding propagation planes, with selected zoomed-in regions. (b) The S_3 Stokes parameters (top) and the relative phase between the circular polarisation components (Φ_{12}) (bottom) for the corresponding propagation planes.

the magnetic filed as:

$$\mathbf{B} = \frac{\mathbf{k}}{c} \times [f_A(\mathbf{r})\mathrm{e}^{i\ell_{\mathrm{A}}\phi}\hat{\sigma}_+ + f_{\mathrm{B}}(\mathbf{r})\mathrm{e}^{i\ell_B\phi}\hat{\sigma}_-], \tag{8}$$

The spin and optical chirality density of a monochromatic beam are given as [4]

$$\mathbf{s} = \mathrm{Im}\frac{\epsilon_0}{2\omega}(\mathbf{E}^* \times \mathbf{E}) \tag{9}$$

and

$$C = -\mathrm{Im}\frac{\omega\epsilon_0}{2}[\mathbf{E}^* \cdot \mathbf{B}], \tag{10}$$

respectively. Inserting Eq. (7) and Eq. (8) gives for the spin and chirality:

$$\mathbf{s} = \frac{\hat{\mathbf{z}}}{c\omega}\Big(I_{\mathrm{A}} - I_{\mathrm{B}}\Big) \tag{11}$$

and

$$C = \frac{\omega}{c^2}\Big(I_{\mathrm{A}} - I_{\mathrm{B}}\Big). \tag{12}$$

Given that the I_{A} represents the intensity of the right CP component and I_{B} left CP, the spin and chirality in a paraxial beam are easily inferred from measuring the third Stokes parameter $S_3 = I_{\mathrm{R}} - I_{\mathrm{L}}$:

$$s_z = \frac{S_3}{\omega c} \tag{13}$$

$$C = \frac{S_3\omega}{c^2} \tag{14}$$

B. Spin and chirality in non-paraxial beams

The longitudinal spin momentum and optical chirality densities for HyOPs framed in terms of non-paraxial Bessel modes (i.e., the radial distribution function is given in terms of Bessel functions of the first kind $f = J_{|\ell|}[k_t r]$) have previously been shown to take the form [5]:

$$\begin{aligned} s_z^E = &\frac{\epsilon_0}{\omega}(J^2_{|\ell_{\mathrm{A}}|}\sin^2\chi - J^2_{|\ell_{\mathrm{B}}|}\cos^2\chi)(1 + \frac{2k_t^2}{k^2}) \\ &+ \frac{k_t^2}{k^2}(J_{|\ell_{\mathrm{A}}|}J_{|\ell_{\mathrm{B}}|\mp2} - J_{|\ell_{\mathrm{B}}|}J_{|\ell_{\mathrm{A}}|\pm2}) \\ &\times \cos[(|\ell_{\mathrm{A}}| - |\ell_{\mathrm{B}}| \pm 2)\phi - 2\theta]\sin 2\chi, \end{aligned} \tag{15}$$

and

$$\begin{aligned} C = &\frac{\epsilon_0\omega}{2c}\bigg[\Big(\frac{k_z}{k} + \frac{k_t^2}{kk_z} + \frac{k_t^2 k_z}{k^3}\Big)(J^2_{|\ell_{\mathrm{A}}|}\sin^2\chi - J^2_{|\ell_{\mathrm{B}}|}\cos^2\chi) \\ &+ \frac{k_t^2}{kk_z}\Big(J^2_{|\ell_{\mathrm{A}}|\pm1}\sin^2\chi - J^2_{|\ell_{\mathrm{B}}|\mp1}\cos^2\chi \\ &+ \Big(\frac{1}{2} + \frac{k_z^2}{2k^2}\Big)\Big(J_{|\ell_{\mathrm{A}}|}J_{|\ell_{\mathrm{B}}|\mp2} - J_{|\ell_{\mathrm{B}}|}J_{|\ell_{\mathrm{A}}|\pm2}\Big) \\ &\times \cos((|\ell_{\mathrm{A}}| - |\ell_{\mathrm{B}}| \pm 2)\phi - 2\theta)\sin 2\chi\Big)\bigg]. \end{aligned} \tag{16}$$

where θ and χ represent the longitude and latitude on the given higher-order Poincare sphere, respectively, and

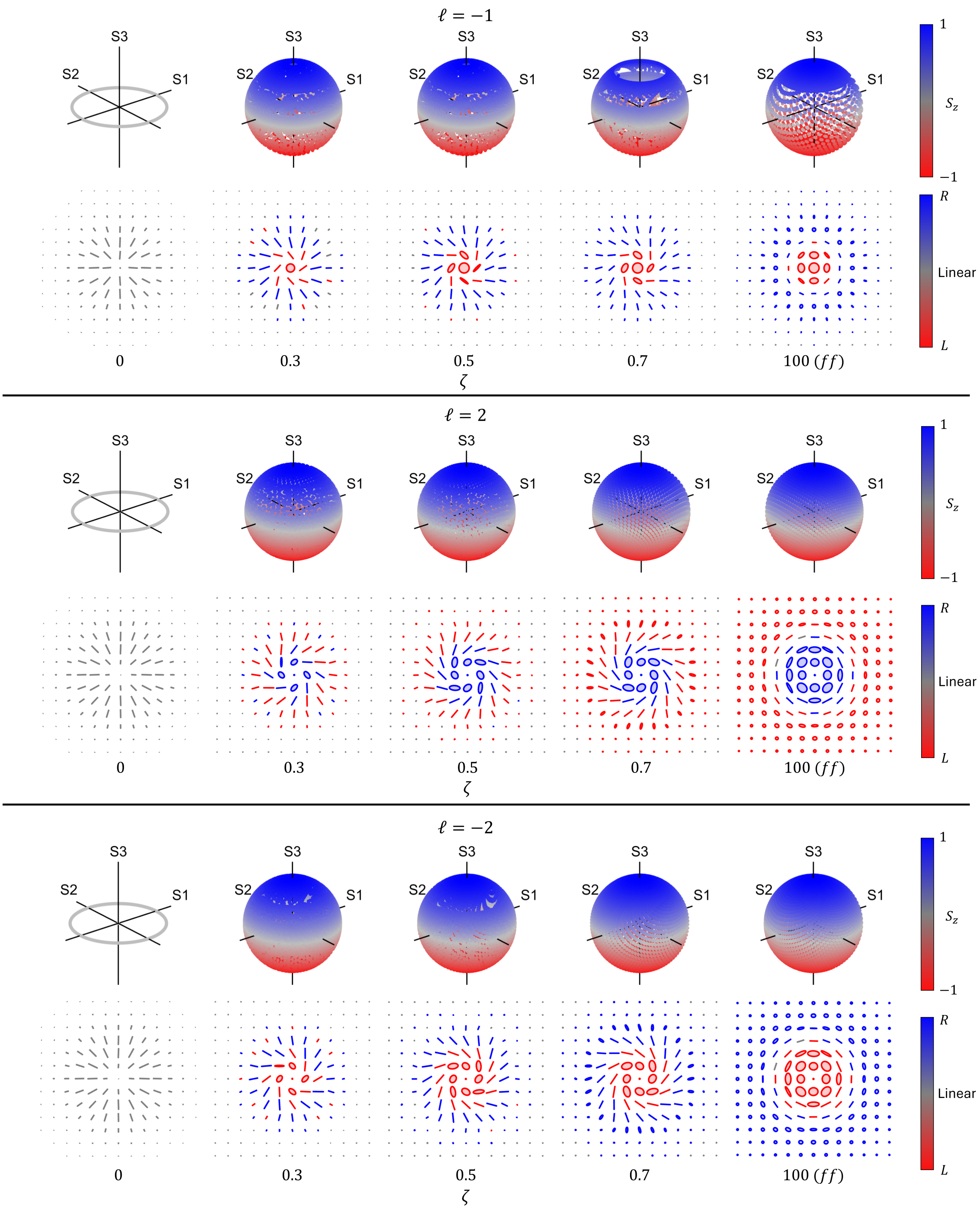

FIG. 4. Simulations for reconstructed sphere coverage and polarization ellipses for $\ell_p = -1$ (top), $\ell_p = 2$ (middle) and $\ell_p = -2$ (bottom) at various propagation planes assuming the entire field intensity can be resolved.

the top sign of $\pm, \mp$ is taken for $\ell_p > 0$ and the bottom sign for $\ell_p < 0$. The paraxial parameter for Bessel beams is k_t/k_z, where k_z is the longitudinal component of the wave vector $\mathbf{k}$ and k_t the transverse component: in a paraxial beam $k_z \approx k$, but as a beam becomes focused the magnitude of k_t grows relative to k_z, and the higher-order (non-paraxial) contributions (i.e. those dependent on k_t) in Eqs. (15) and (18) become important.

The radially polarized CVVBs used in our work are described by the angles $\chi = \pi/4$ and $\theta = 0$. We study $\eta = 1$ input beams with the q-plate converting $|L\rangle$ into $|R\rangle$ and vice-versa, while introducing a phase of $\mathrm{e}^{i(\ell_p - 1)\phi}$ and $\mathrm{e}^{i(\ell_p + 1)\phi}$ to $|R\rangle$ and $|L\rangle$, respectively This means we are restricted to integer values of the Pancharatnam charge, such that $\pm\ell_p = \pm(|\ell_\mathrm{A}|+|\ell_\mathrm{B}|)/2$, and polarisation index $\mp\eta = \pm(|\ell_\mathrm{B}| - |\ell_\mathrm{A}|)/2)$.

As an example $\ell_p = 1$ would be constructured from $\ell_\mathrm{A} = 0$ and $\ell_\mathrm{B} = 2$ which gives a spin and chirality density:

$$s_z^E = \frac{\epsilon_0}{\omega}(J_0^2 - J_2^2)\Big(\frac{1}{2} + \frac{2k_t^2}{k^2}\Big) \tag{17}$$

Which is simply the paraxial result plus a very small non-paraxial correction which has an identical spatial distribution. This highlights that even under non-paraxial conditions the paraxial contribution is still the most important generator of spin in OILS of vector vortex beams. The chirality is

$$C = \frac{\epsilon_0\omega}{2c}\Big[(J_0^2 - J_2^2)\Big(\frac{k_z}{2k} + \frac{k_t^2}{kk_z} + \frac{k_t^2 k_z}{k^3}\Big)\Big]. \tag{18}$$

Similar to the spin, we find that the non-paraxial contributions have identical spatial distributions to the paraxial chirality and that the paraxial contribution is the key generator of chirality in vector vortex beams. In a paraxial beam $k_z \approx k$ and we see that $s_z \propto C$. Similar analysis is simple to carry out for other values of ℓ_p we used in our experiments. All highlight the fact that in vector vortex beams OILS is dominated by paraxial optics.

[1] Vasilios Cocotos, Light Mkhumbuza, Kayn A Forbes, Robert de Mello Koch, Angela Dudley, and Isaac Nape. Laguerre–gaussian modes become elegant after an azimuthal phase modulation. *Optics Letters*, 50(6):1913–1916, 2025.

[2] S Saghafi and CJR Sheppard. Near field and far field of elegant hermite-gaussian and laguerre-gaussian modes. *Journal of Modern Optics*, 45(10):1999–2009, 1998.

[3] Avi Niv, Gabriel Biener, Vladimir Kleiner, and Erez Hasman. Manipulation of the pancharatnam phase in vectorial vortices. *Optics Express*, 14(10):4208–4220, 2006.

[4] Konstantin Y Bliokh, Aleksandr Y Bekshaev, and Franco Nori. Dual electromagnetism: helicity, spin, momentum and angular momentum. *New Journal of Physics*, 15(3):033026, 2013.

[5] Kayn A Forbes. Spin angular momentum and optical chirality of poincaré vector vortex beams. *Journal of Optics*, 26(12):125401, 2024.

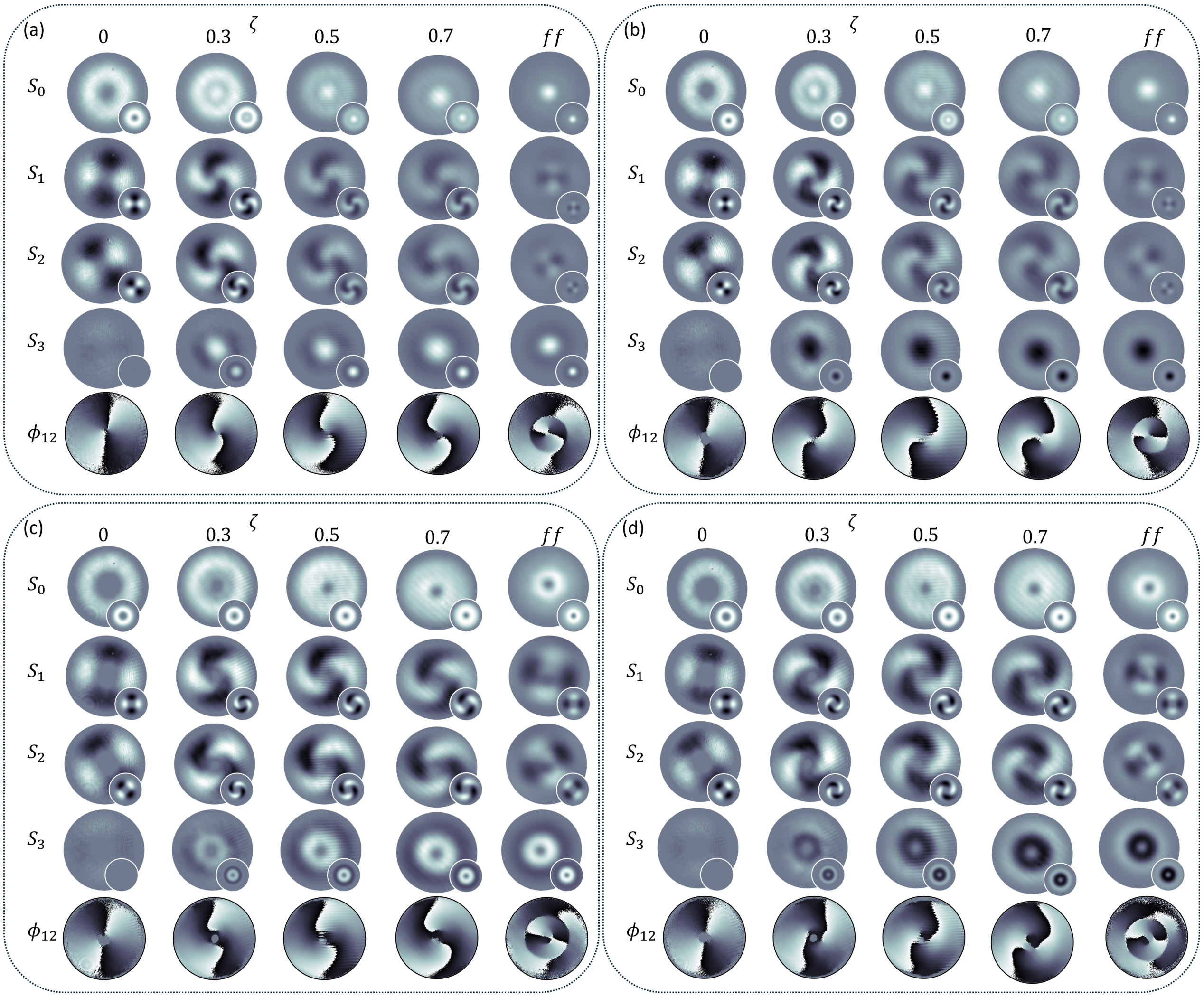

FIG. 5. Schematic showing all stokes parameters at different propagation planes: **(a)** $\ell_p = 1$, **(b)** $\ell_p = -1$, **(c)** $\ell_p = 2$ **and (d)** $\ell_p = -2$
